# Comprehensive characteristics of pathological subtypes in testicular germ cell tumor: Gene expression, mutation and alternative splicing

**DOI:** 10.3389/fimmu.2022.1096494

**Published:** 2023-01-13

**Authors:** Xiangyang Yao, Hui Zhou, Chen Duan, Xiaoliang Wu, Bo Li, Haoran Liu, Yangjun Zhang

**Affiliations:** ^1^ Department of Urology, Zhongnan Hospital of Wuhan University, Wuhan, China; ^2^ Department of Urology, Tongji Hospital, Tongji Medical College, Huazhong University of Science and Technology, Wuhan, China; ^3^ Stanford Bio-X, Stanford University, Stanford, CA, United States; ^4^ Cancer Precision Diagnosis and Treatment and Translational Medicine Hubei Engineering Research Center, Zhongnan Hospital of Wuhan University, Wuhan, China; ^5^ Department of Biological Repositories, Zhongnan Hospital of Wuhan University, Wuhan, China

**Keywords:** testicular germ cell tumor, gene expression, alternative splicing, subtype, mutation

## Abstract

**Background:**

Testicular germ cell tumor (TGCT) is the most common tumor in young men, but molecular signatures, especially the alternative splicing (AS) between its subtypes have not yet been explored.

**Methods:**

To investigate the differences between TGCT subtypes, we comprehensively analyzed the data of gene expression, alternative splicing (AS), and somatic mutation in TGCT patients from the TCGA database. The gene ontology (GO) enrichment analyses were used to explore the function of differentially expressed genes and spliced genes respectively, and Spearman correlation analysis was performed to explore the correlation between differential genes and AS events. In addition, the possible patterns in which AS regulates gene expression were elaborated by the ensemble database transcript atlas. And, we identified important transcription factors that regulate gene expression and AS and functionally validated them in TGCT cell lines.

**Results:**

We found significant differences between expression and AS in embryonal carcinoma and seminoma, while mixed cell tumors were in between. GO enrichment analyses revealed that both differentially expressed and spliced genes were enriched in transcriptional regulatory pathways, and obvious correlation between expression and AS events was determined. By analyzing the transcript map and the sites where splicing occurs, we have demonstrated that AS regulates gene expression in a variety of ways. We further identified two pivot AS-related molecules (SOX2 and HDAC9) involved in AS regulation, which were validated in embryonal carcinoma and seminoma cell lines. Differences in somatic mutations between subtypes are also of concern, with our results suggesting that mutations in some genes (B3GNT8, CAPN7, FAT4, GRK1, TACC2, and TRAM1L1) occur only in embryonal carcinoma, while mutations in KIT, KARS, and NRAS are observed only in seminoma.

**Conclusions:**

In conclusion, our analysis revealed the differences in gene expression, AS and somatic mutation among TGCT subtypes, providing a molecular basis for clinical diagnosis and precise therapy of TGCT patients.

## Introduction

Testicular germ cell tumor (TGCT) accounts for 1% of newly diagnosed tumors in men (especially men aged 20-40 years) and 5% of tumors of the urogenital tracts worldwide (1). The incidence of TGCT varies significantly between different regions and ethnic groups, with high incidence in developed European countries such as Denmark and relatively low incidence in low-income areas such as Asia and Africa, suggesting that genetic and environmental factors play an essential role in the development of cancer (2). TGCT can be histologically divided into two subtypes: seminoma and non-seminoma. As the primary subtype, seminoma accounted for 95% of the whole TGCT, while non-seminoma (including four types: embryonal carcinoma, yolk sac tumor, teratoma, and mixed germ cell tumor) has a small proportion, but prone to metastasis resulting in poor prognosis (3, 4).

Several multidisciplinary therapies, such as surgery combined with chemoradiotherapy, have significantly improved the prognostic survival of TGCT patients over the past few decades (5). Recent studies on the resistance of platinum-based chemotherapeutics have been continuously reported. By using TGCT cell lines, Caggiano et al. revealed that TGCT achieved cisplatin resistance through the regulation of DNA repair enzymes, and noted that PARP inhibitors combined with low dose cisplatin could reduce toxic reactions (6). Using a model of cisplatin resistance, Fazal et al. indicated that the overall remodeling of DNA methylation is a key factor in drug resistance (7). About 30% of patients have unsatisfactory treatment or resistant cisplatin, and different subtypes have different manifestations of this resistance (8). Different responses to treatment between subtypes may be attributed to significant differences in expression profiles and molecular mechanisms (9, 10). Previous analysis of macroarray studies found that differentially expressed genes were predominantly upregulated in seminoma, while down-regulated in non-seminoma, and showed activation differences in DNA synthesis, DNA repair, and cell proliferation (11). Sun et al. revealed the features of non-seminoma metastasis and poor prognosis by analyzing telomere length between TGCT subtypes (12). Furthermore, the epigenetics (specifically DNA methylation) of TGCTs was also analyzed, and DNA methylation-based models and biomarkers were identified that may provide the basis for better prognosis and treatment of TGCT (13, 14). Shen et al. primarily elucidate differences in somatic mutation and DNA methylation between TGCT subtypes through multi-omics analysis (15). However, the differences in alternative splicing between different TGCT subtypes have not been explored, and the relationship between expression and splicing remains to be elucidated. Therefore, it is urgent to understand the differences between the molecular basis and biological functions of the various subtypes, to provide new ideas for the carcinogenic mechanism, clinical diagnosis and treatment of TGCT.

In this study, we comprehensively analyzed the differences in gene expression, alternative splicing (AS), and somatic mutations between TGCT subtypes. Subtype differences were observed mainly between seminoma and embryonal carcinoma. In addition, the correlation between AS and expression was analyzed. Finally, two key genes regulating transcription and AS, SOX2 and HDAC9, were identified for experimental verification.

## Materials and methods

### Data acquisition and processing

The gene expression data for RNA-seq (n=156, platform: Illumina HiSeq), somatic mutation data (n=144, platform: Illumina-MuTect2) and clinical data (n=164) were downloaded from GDC Testicular Cancer (TGCT) cohort of TCGA data portal (https://xenabrowser.net). Splicing data (n=149) of TGCT was obtained from TCGA SpliceSeq database (http://bioinformatics.mdanderson.org/TCGASpliceSeq/index.jsp). As a quantitative indicator of alternative splicing, the Percent Spliced In (PSI) value represents the ratio of inclusion and exclusion of different exons.

We carefully reviewed and excluded 25 samples with no clinical information and subtypes with too small sample size in TGCT (5 cases of teratocarcinoma, 6 cases of benign teratocarcinoma, and 4 cases of yolk sac tumor), and finally obtained 124 patients clinical characteristics (**Table 1**). Subsequently, we integrated the RNA-seq data, splicing data and somatic mutation data with the clinical information to obtain the expression, splicing and mutation profiles of 120, 120 and 114 patients, respectively.

**Table 1:** Characteristics of subtypes with TGCT based on TCGA.

Characteristics	Embryonal carcinoma n/26 (%)	Mixed germ cell tumor n/30 (%)	Seminoma n/68 (%)	Sum (%) (n=124)
Age				
<=30	16/26 (61.5)	18/30 (60.0)	25/68 (36.7)	59 (47.6)
>30	10/26 (38.5)	12/30 (40.0)	43/68 (63.3)	65 (52.4)
P(Fisher)	0.1238	0.1127	0.0095	
Topography (T)				
T1	7/26 (26.9)	18/30 (60.0)	42/68 (61.8)	67 (54.0)
T2-T3	19/26 (73.1)	12/30 (40.0)	25/68 (36.7)	56 (45.2)
Tx	0/26 (0.0)	0/30 (0.0)	1/68 (1.5)	1 (0.8)
P(Fisher)	0.0033	0.7138	0.0253	
Lymph node (N)				
N0	7/26 (26.9)	13/30 (43.3)	22/68 (32.4)	42 (33.8)
N1-N2	7/26 (26.9)	3/30 (10.0)	3/68 (4.4)	13 (10.5)
Nx	12/26 (46.2)	14/30 (46.7)	43/68 (63.2)	69 (55.7)
P(Fisher)	0.0273	0.1773	0.1026	
Metastasis (M)				
M0	22/26 (84.6)	24/30 (80.0)	61/68 (89.7)	107 (86.3)
M1	1/26 (3.9)	3/30 (10.0)	0/68 (0.0)	4 (3.2)
Mx	3/26 (11.5)	3/30 (10.0)	7/68 (10.3)	13 (10.5)
P(Fisher)	1	0.0310	0.0377	
Stage				
Stage I	10/26 (38.5)	15/30 (50.0)	52/68 (76.5)	77 (62.1)
Stage II-III	15/26 (57.7)	13/30 (43.3)	14/68 (20.6)	42 (33.9)
Not reported	1/26 (3.8)	2/30 (7.2)	2/68 (2.9)	5 (4.0)
P(Fisher)	0.0092	0.1125	0.0004	
Serum markers				
S0	8/26 (30.8)	3/30 (10.0)	29/68 (42.6)	40 (32.3)
S1-S3	17/26 (65.4)	27/30 (90.0)	26/68 (38.2)	70 (56.5)
Sx	1/26 (3.8)	0/30 (0.0)	13/68 (19.2)	14 (11.3)
P(Fisher)	0.6381	0.0005	0.0006	

Fisher’s exact test was performed between patients in each TGCT subtype and patients in other TGCT subtypes for each clinical characteristic. P<0.05 was considered significant.

### Differential analysis of mRNA expression and alternative splicing

One-way analysis of variance (ANOVA) of gene expression in the three main types of TGCT (embryonal carcinoma, mixed germ cell tumor, and seminoma) revealed the most significant differences between embryonal carcinoma and seminoma. The R package “limma” was used to identify differential expression genes (DEGs) between the two subtypes (FDR<0.05, logFC>1). Due to the distribution of PSI and the ratio of splicing events, Kruskal-Wallis test was first used to analyze the differences of alternative splicing among the three groups. Wilcoxon rank-sum test and delta median PSI value between embryonal carcinoma and seminoma were used to identify differential splicing events (Benjamini & Hochberg adjusted *P*<0.05, delta PSI > 0.1) (16).

### Analysis of somatic mutation profile

The 3199 somatic mutation data of 144 TGCT samples downloaded from TCGA were annotated with gencode.v22.annotation and merged with processed clinical data to obtain 2435 mutation data of 114 patients. Given that there are multiple descriptions of a mutation type, we sorted out the types of mutation. For example, we grouped the “inframe deletion” and “inframe insertion” into the “inframe variants”, and obtained five main types of mutation (including frameshift variant, inframe variant, missense variant, splice site variant and stop gained). In addition, the mutation frequency of each gene in the three types of TGCT was calculated, and the genes with mutations in more than 5% of the individuals were identified, which were considered high frequency mutation genes. A total of 21 significantly mutated genes with a mutation frequency greater than 1% were identified. Finally, we calculated and visualized the mutation frequency of these genes as a whole and the mutation percentage within their respective subtypes. In addition, the cBioPortal website (http://www.cbioportal.org/) was used to analyze mutation sites of the significantly high-frequency mutation genes (17, 18).

### Gene ontology enrichment analysis

The Database for Annotation, Visualization and Integrated Discovery (DAVID, version 6.8, https://david.ncifcrf.gov/) was used to identify enriched pathways, and pathways with FDR<0.05 were considered significant (19).

### Cell lines and cell culture

The human TGCT cell lines TCam2 (a seminoma-like cell line, RRID: CVCL_T012) and NCCIT (an embryonic-like cell line, RRID: CVCL_1451) were purchased from the Shanghai Cell Bank Type Culture Collection Committee (Shanghai, China). TCam2 cells were cultured in RPMI 1640 medium and NCCIT cells were grown in DMEM, both of which contain 10% fetal bovine serum (FBS) and maintained in an incubator at 37°C and 5% CO2.

### Plasmids and stable transfected cells establishment

The target fragments were inserted into lentiviral vectors such as pCDH-MSCV-MCS-EF1-copGFP (RRID: Addgene_72266), all plasmids were verified by DNA sequencing. The recombinant lentiviral vectors were transfected into HEK293 (RRID: CVCL_0045) cells together with pGC-LV, pHelper 1.0 and pHelper 2.0 plasmids and incubated for 48-72h. The viral fluid was then collected to infect target cells for 3 days and treated with puromycin for 14 days. After confirming the efficiency of the interfering plasmid by PCR, the surviving cells were used for further experiments.

### Quantification of gene expression and alternative splicing

Total RNA was extracted using TRIzol reagent (Thermo Fisher Scientific, USA) for TGCT cell lines. cDNA synthesis was obtained by reverse transcription PCR (RT-PCR) using 5×HiScript III RT SuperMix (Vazyme, Nanjing, China). Semi-quatitative PCR (semi-qPCR) was performed with 2×Green PCR Mix (Vazyme, Nanjing, China) using thermal cyclers (Applied Biosystem, American). Quantitative real-time PCR (qRT-PCR) was performed with 2×ChamQ Universal SYBR qPCR Master Mix* (Vazyme, China) through QuantStudio Real-Time PCR (Applied Biosystem, American). Splicing specific transcripts were distinguished using agarose gel electrophoresis and grayscale-measured using software Image J (RRID : SCR_003070). All primers were provided in the **Supplementary Table S1**.

### Colony formation assay

Cells were digested into a single cell suspension and plated at 1×10^3^ cells per well in six-well plates for 14-21 days. Then the cell colonies were stained with crystal violet and calculated the number of cell colonies when the cells grew into visible colonies.

### CCK-8 assay

In the CCK-8 assay, cells were seeded into 96-well plates at 2×10^3^ cells per well. After adding 10 μL of CCK-8 to each well and incubating for 2 hours, the absorbance of each well was measured at a wavelength of 450 nm.

### Transwell migration assay

About 5 × 10^4^ cells were digested into a single-cell suspension and seeded in the upper chamber of a 24-well Transwell plate containing 200 μL of FBS-free medium. Then, 500 μL of target cell culture medium with 10% FBS was added to the lower chamber. After 12 hours of incubation, they were fixed with methanol and stained with crystal violet for 30 minutes. Finally, imaging and counting were performed under an inverted microscope.

### Statistical analysis and visualization

All experiments were repeated in three times and data are presented as mean ± SD. Comparisons between two groups were performed using Student’s t test. *P*<0.05 was considered significant. All statistical analyses were performed with R software (version 4.0.3) and GraphPad Prism (RRID : SCR_002798). In data processing and visualization with R software, R packages such as limma, edgeR, ggplot2, ggrepel, ggpubr, clusterProfiler, stringr, ComplexHeatmap, UpSetR and VennDiagram were used.

## Results

### Clinical characteristics of TGCT subtypes

To investigate the differences between the major pathological types of TGCT, we analyzed clinical data of 124 patients downloaded from TCGA, including embryonal carcinoma (n = 26), mixed germ cell tumor (30), and seminoma (68). The clinical characteristics of the patients were shown in **Table 1**. Overall, the number of patients younger than 30 and older than 30 were almost equal. However, within the subtypes, embryonal carcinoma and mixed germ cell tumor accounted for most patients younger than 30 years old (61.5%, 60.0%, respectively), while seminoma has the opposite effect, with the majority of patients over 30 years of age (63.3%, P=0.0095). In the T classification, the performance of the three subtypes is completely different. T2-T3 accounted for more than two-thirds (73.1%, P=0.0033) of embryonal carcinoma, while seminoma was dominated by T1 (61.8%, P=0.0253), and mixed germ cell tumors were in between. In the N classification, the proportion of Nx (the regional lymphatic metastasis cannot be estimated) accounted for more than half (55.7%), making it impossible to compare the differences between subtypes. In the M (metastasis) classification, the proportion of M0 (no metastasis) accounted for the vast majority of the three types (84.6%, 80.0%, 89.7%, respectively), indicating that TGCT rarely occurs distant metastasis. Among the three types, mixed germ cell tumor has the relatively highest degree of metastasis. As for staging, we observed a similar result to the T classification. Stage II and III was predominantly in embryonal carcinoma (57.7%, P=0.0092), stage I of seminoma was more than three-quarters (76.5%, 0.0004). Among serum markers, the proportion of S1-S3 in mixed germ cell tumors was the highest (90.0%, P=0.0005), while in seminoma was the lowest (38.2%, P=0.0006).

### Differences of gene expression between embryonal carcinoma, mixed germ cell tumor and seminoma

**Figure 1:**
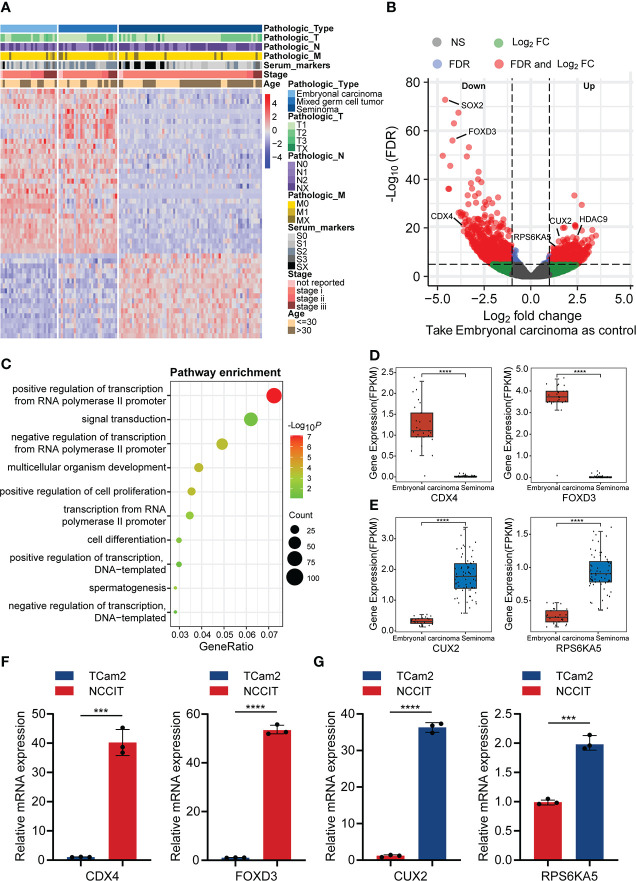
The difference in gene expression between TGCT subtypes. **(A)** Heatmap showed the difference in expression of the top 1000 genes between embryonal carcinoma, mixed germ cell tumor and seminoma analyzed by ANOVA (FDR<0.05). **(B)** The volcano plot demonstrated the differentially expressed genes between embryonal carcinoma and seminoma analyzed by R package “limma” (FDR<0.05, logFC>1). Up: Up-regulated differential genes in seminoma compared with embryonal carcinoma; Down: Down-regulated differential genes in seminoma compared with embryonal carcinoma. **(C)** The bubble chart indicated the GO enrichment of the differential genes between embryonal carcinoma and seminoma. **(D, E)** The boxplot revealed the expression of the two groups of differential genes (data from TCGA). **(F, G)** Expression of the above genes was verified by qPCR in the TGCT cell lines. ANOVA: one-way analysis of variance. ****P < 1e-04, ***P < 0.001.

To explore expression differences between the main pathological types of TGCT, we analyzed the expression data of 120 patients with TGCT (including embryonal carcinoma, mixed germ cell tumor and seminoma) from TCGA. Through one-way analysis of variance (ANOVA), we found that 13675 (FDR<0.0001) of the total 58367 genes have differences among the three subtypes. Due to the large amounts of different genes, we selected the first 1000 genes with significant differences for visualization (**Figure 1A**). Interestingly, the difference between embryonal carcinoma and seminoma was the most significant among the three subtypes, while mixed germ cell tumor lied between them, which was consistent with the pathological classification of TGCT. Therefore, we further analyzed the differences between embryonal carcinoma and seminoma using R package “limma”, and identified 2079 differential expression genes (DEGs) (FDR<0.05, logFC>1), including 869 genes up-regulated and 1210 genes down-regulated in seminoma when compared with embryonal carcinoma. (**Figure 1B**). To understand whether the function of the DEGs is related to subtypes, we performed GO enrichment analysis. The results indicated that DEGs were highly enriched in “positive/negative regulation of transcription from RNA polymerase II promoter”, “signal transduction”, “positive regulation of cell proliferation” and other pathways (**Figure 1C**). In addition, we showed the number of up-regulated and down-regulated genes in each pathway (**Supplementary Figure S1A**). Cell proliferation is a key step in the process of tumor cell migration. Heatmap analysis of the enrichment genes in “positive regulation of cell proliferation” pathway demonstrated that more than 80% of the genes were expressed higher in embryonal carcinoma than in seminoma, which also provided side evidence for the higher malignancy and poor prognosis of embryonal carcinoma in clinical practice (**Supplementary Figure S2A**).

In addition, several clinically relevant genes were selected for independent analysis to show the specific conditions of DEGs in the two subtypes. Teratocarcinoma-derived growth factor1 (TDGF-1) from the epidermal growth factor family (EGF), is a glycoprotein that plays a crucial regulatory role in the migration, induction, differentiation and signal transduction of embryonic stem cells (20). Baldassarre and his colleagues pointed out that TDGF-1 was highly expressed in 100% of non-seminoma (including embryonal carcinoma), but only 30% of seminoma, and this result was similar with ours (**Supplementary Figure S2B**) (21). TNFRSF8/CD30 was expressed in most embryonal carcinoma, whereas in only about 10% of seminoma (22), and was validated in our results (**Supplementary Figure S2C**). However, some other genes appeared relative higher expression in seminoma. Melanoma-associated gene C2 (MAGEC2) is a cancer-testis antigen expressed in testicular tissues. Using tissue microarray analysis, bode et al. discovered that MAGEC2 was significantly overexpressed in seminoma and may be a reliable marker for distinguishing seminoma and embryonal carcinoma (**Supplementary Figure S2D**) (23). L-type amino acid transporter 3 (LAT3/SLC43A1), a vital transporter the uptake of amino acids, is expressed abundantly in seminoma, which may cause the difference between seminoma and embryonal carcinoma (**Supplementary Figure S2E**) (24). We analyzed the molecules that have received more clinical attention above, and considered whether exist molecular targets that are important for the classification of TGCT subtypes but have not yet been discovered. Caudal Type Homeobox 4 (CDX4), a member of the small subfamily of homeobox-containing transcription factors, affects adult acute lymphoblastic leukemia by regulating HOX gene expression (25, 26). Similarly, Forkhead Box D3 (FOXD3) from the forkhead family of transcription factors is a key transcriptional repressor. FOXD3 has been found to promote apoptosis in colorectal cancer and non-small cell lung cancer cells through its transcriptional repression (27, 28). Our analysis suggested that the expression of CDX4 and FOXD3 from TGCT cohort was significantly higher in embryonal carcinoma than in seminoma (**Figure 1D**), which was verified by qRT-PCR experiments (**Figure 1F**). In addition, the protein encoded by Cut Like Homeobox 2 (CUX2) is a cofactor for DNA damage and repair, and the role of CUT domain proteins in DNA repair is exploited by cancer cells to promote their survival (29). Studies have shown that CUX2 is highly expressed in thyroid cancer and promotes tumor cell invasion and migration (30). Ribosomal Protein S6 Kinase A5 (RPS6KA5) activates ATP-binding activity and protein serine/threonine kinase activity by participating in multiple histone serine phosphorylation and transcriptional regulation. It has been reported that RPS6KA5 can act as a tumor-associated antigen (TAAs) in lung cancer to distinguish lung cancer patients from healthy people (31). We found that CUX2 and RPS6KA5 were significantly overexpressed in the TGCT cohort of seminoma (**Figure 1E**), and the same results were obtained by quantitative PCR (**Figure 1G**). Here we only show a small part of the genes that have received attention, and there are many potential DEGs worth exploring for their clinical value across TGCT subtypes.

### Differences of alternative splicing between embryonal carcinoma, mixed germ cell tumor and seminoma

**Figure 2:**
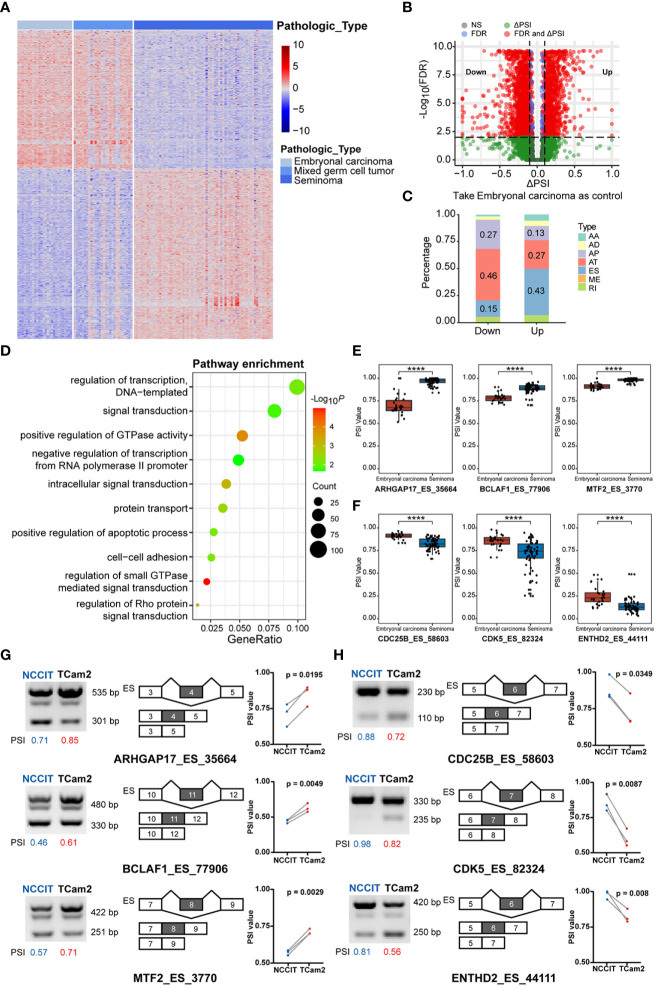
The difference in alternative splicing (AS) between TGCT subgroups. **(A)** Kruskal-Wallis test was performed to analyze the difference in AS events between embryonal carcinoma, mixed germ cell tumor and seminoma (top 1000 events, FDR<0.05). **(B)** Wilcoxon rank-sum test and delta median PSI value was used to analyze the difference between embryonal carcinoma and seminoma (FDR<0.05, delta PSI>0.1). Take embryonal carcinoma as control. Up: Splicing events with higher PSI values in seminoma than in embryonal carcinoma; Down: Splice events with lower PSI values in seminoma than in embryonal carcinoma. **(C)** The percentage bar graph showed the difference in splicing types between the up and down groups. **(D)** GO term enrichment of differentially spliced (DAS) genes. **(E, F)** Comparison of PSI values for differential AS events in two subtypes. **(G, H)** Differential expression of spliced transcripts was validated in the TGCT cell lines through semi-qPCR and corresponding agarose gel electrophoresis. Calculate the PSI of each lane by dividing the gray value of the longer transcript by the sum of the gray value of the longer and shorter transcripts. ****P < 1e-04.

Integrating the pathological types of TGCT with alternative splicing (AS) events, 42415 AS events of 120 patients were included in our study. Containing seven main AS types: alternate acceptor site (AA, 3441), alternate donor site (AD, 2992), alternate promoter (AP, 8413), alternate terminator (AT, 8721), exon skip (ES, 15879), retained intron (RI, 2790), and mutually exclusive exons (ME, 179)(**Supplementary Figure S3A**) (32). The number of seven splicing types of all AS events were shown in **Supplementary Figure S3B**. To explore the relationship between AS events and TGCT subtypes, we used the Kruskal-Wallis test to analyze the PSI values of all AS events, and found that 4985 splicing events were different between the three subtypes (FDR<0.0001). The heatmap of the first 1000 significant splicing events was shown in **Figure 2A**. Similar to the results of gene expression, significant differences also existed in AS events between embryonal carcinoma and seminoma. Wilcoxon rank-sum test was further performed for all AS events between the two subtypes, and 2704 differential alternative splicing events (DASEs) were identified with FDR<0.05 and absolute delta PSI >0.1, including 1096 DASEs with up-regulated PSI and 1608 DASEs with down-regulated PSI in seminoma (**Figure 2B**). Moreover, the difference also exists in the percentage of splicing types for PSI value in embryonal carcinoma or seminoma. The results indicated that, in seminoma, AT was the predominant splicing type in the splicing events with significantly decreased PSI values, while ES was the dominated with significantly increased PSI values using embryonal carcinoma as a control (**Figure 2C**). Considering that function of differentially spliced genes may affect the subtype classification, GO enrichment analysis was done to describe enriched pathways of these genes. The results showed that these differentially spliced genes were significantly enriched in pathways such as “regulation of transcription, DNA−templated”, “signal transduction”, “positive regulation of GTPase activity”, and “negative regulation of transcription from RNA polymerase II promoter” (**Figure 2D**). Moreover, the number of up-regulated and down-regulated splicing genes in each pathway were demonstrated in **Supplementary Figure S1B**. The biological processes related to the regulation of GTPase activity play an important role in the cell cycle and energy metabolism of tumor cells. Rho GTPase Activating Protein 17 (ARHGAP17), a member of the GTP-active Protein family, inhibits tumor progression by down-regulating the PI3K/Akt pathway (33). The PSI value of ARHGAP17 was significantly upregulated in seminoma and confirmed by PCR in TCam2 cells (**Figures 2E, G** **)**. BCL2 Associated Transcription Factor 1 (BCLAF1) encodes a transcriptional repressor that interacts with the BCL2 protein family to regulate apoptosis. Studies have shown that alternative splicing of exon 5 of BCLAF1 produces two splice isoforms, and the longer splice isoform is prevalent in colon cancer and promotes tumor proliferation (34). Interestingly, our analysis found that the long isoform of BCLAF1 with exon 11 included was upregulated in seminoma and validated in TCam2 cells (**Figures 2E, G** **)**. Metal Response Element Binding Transcription Factor 2 (MTF2) is involved in the regulation of histone methylation and the transcriptional regulation of RNA polymerase II. Studies have found that MTF2, as an accessory subunit of PRC2, is an important target for the treatment and prevention of myeloma (35). In seminoma, the PSI value of MTF2 was found to be significantly upregulated and consistent with the experimental results (**Figures 2E, G** **)**. Abnormal cell cycle is one of the basic mechanisms of tumorigenesis, making the regulation of cell cycle mechanism a reasonable target for anticancer therapy (36). Cell Division Cycle 25B (CDC25B) participates in the regulation of mitosis by activating the cyclin-dependent kinase CDC2. CDC25B is overexpressed in tumor cells and is an important driver of various cancers (37–39). We found that the PSI value of CDC25B was upregulated and significantly different in embryonal carcinomas, which consistent with the experimental results in NCCIT cells (**Figures 2F, H** **)**. Cyclin Dependent Kinase 5 (CDK5) is also a key molecule in regulating cell cycle, and downregulation of CDK5 can inhibit the expression of PDL1 and promote anti-tumor immunity (40). In addition, CDK5 produces multiple splicing isoforms, and our results showed that the PSI value of the long splice isoform of CDK5 exon 6 is significantly upregulated in embryonal carcinoma, and validated in NCCIT cells (**Figures 2F, H** **)**. TEPSIN Adaptor Related Protein Complex 4 Accessory Protein (TEPSIN) is a membrane serine protease expressed in a variety of human tissues including kidney, prostate and thyroid (41), precise control of Hepsin proteolytic activity is an effective treatment for prostate cancer (42). Less reported about its splicing role, we found that the PSI value of TEPSIN was significantly up-regulated in embryonal carcinoma and NCCIT cells (**Figures 2F, H** **)**.

### Regulation of gene expression by alternative splicing

**Figure 3:**
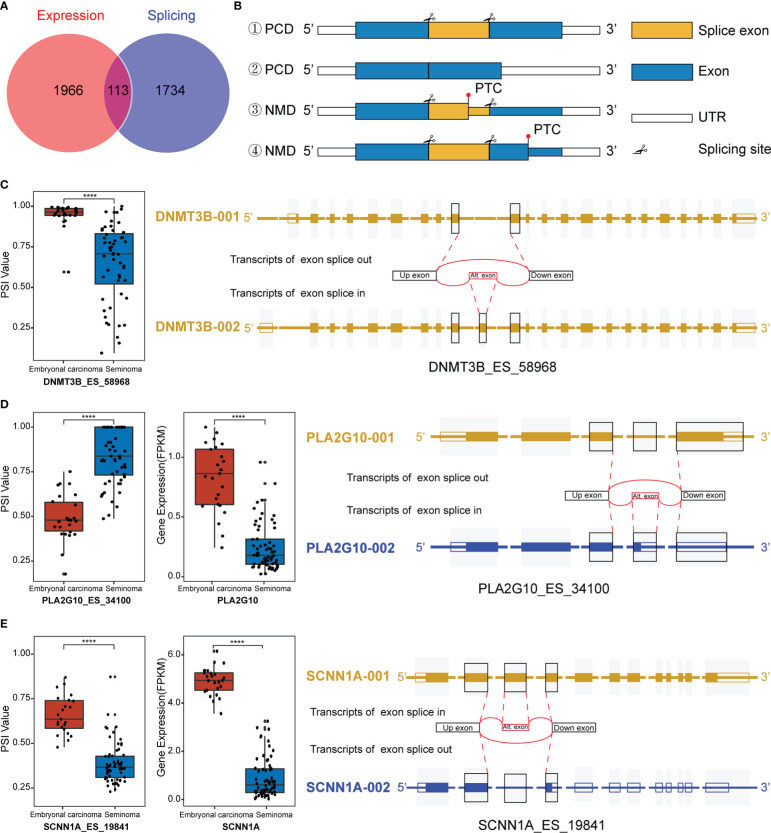
Regulation of transcripts by alternative splicing (AS). **(A)** Venn diagram of differentially spliced (DAS) and differentially expressed genes (DEGs). **(B)** Maps of transcript length regulated by alternative splicing which happened at different sites. #1 and #2 are long and short isoforms encoding proteins after exon splicing, while #3 and #4 are NMD with premature termination of transcription. PCD: Protein coding. NMD: Nonsense-mediated mRNA decay. PTC: Premature termination codon. UTR: Untranslated regions. **(C)** Splicing map of the splicing-expression gene DNMT3B. **(D, E)** Differentially expressed and spliced profile of PA2AG10 and SCNN1A in the two subtypes of TGCT, and the transcriptional maps obtained from the Ensemble website. ****P < 1e-04.

Interestingly, we observed that differentially spliced genes and expressed genes were both enriched in the “negative regulation of transcription from RNA polymerase II promoter” pathways. Given the multifaceted impact of AS on transcripts, we hypothesized that AS may regulate function and expression of genes in these pathways. Combining the DEGs and genes related to DASEs between the two groups, a total of 113 splicing-expression related genes were identified, followed by a comprehensive analysis of the splicing patterns of these genes (**Figure 3A**). There are various types of alternative splicing, only exon skipping is exemplified. It is well known that AS produces long and short splicing-​​isoforms (the first two cases in **Figure 3B**), but this splicing may not necessarily affect protein coding (PCD) transcript degradation. DNA Methyltransferase 3 Beta (DNMT3B) encodes DNA Methyltransferase, which is essential for methylation modification and embryonal development, and DNMT3B PSI values were observed to be significantly elevated in embryonal carcinoma (**Figure 3C**). To explore specific changes in transcripts, we downloaded the transcriptional map of DNMT3B from the ensemble genome database (43). It was found that there was no change in exons except ES in exon 11 between transcripts 1 and 2, and the transcript was not degraded, indicating that the AS of exon 11 of DNMT3B only affected the ratio of long and short splicing isoforms (**Figure 3C**). Of course, AS also regulates gene expression by mediating a decrease in the ratio of PCD transcripts, while a significant increase in the ratio of nonsense-mediated mRNA decay (NMD) transcripts. In the third case in **Figure 3B**, the ES transcript contains a premature termination codon (PTC), thus leading to premature termination of transcription to form NMD. Phospholipase A2 Group X (PLA2G10) a member of the Phospholipase A2 family that encodes Phospholipase A2 as a predictive marker for non-small cell lung cancer, where alternative splicing leads to multiple transcriptional variants (44). After the splicing of exon 4 on the long transcript, the transcript was degraded due to the early termination of transcription, which ultimately promoted the decrease of expression (**Figure 3D**). In addition, ES may induce PTC in downstream exons, thus promoting the formation of NMD. Sodium Channel Epithelial 1 Subunit Alpha (SCNN1A) encodes one of the subunits of sodium channels involved in the transport of fluid and electrolytes in epithelial cells. Studies have shown that SCNN1A is overexpressed in ovarian cancer and promotes cell proliferation, migration and predicts poor prognosis by regulating epithelial-mesenchymal transition (45). We observed a significant decrease in the expression of SCNN1A in seminoma, probably due to the occurrence of PTC in downstream exons caused by AS in exon 3, which ultimately affected the levels of its mRNA transcripts (**Figure 3E**). Although most of what we have observed clinically are differences in gene expression between the subtypes, the reason for this difference is what we are really concerned about, and AS is one of the most important reasons.

### Regulation of alternative splicing by transcription factors

**Figure 4:**
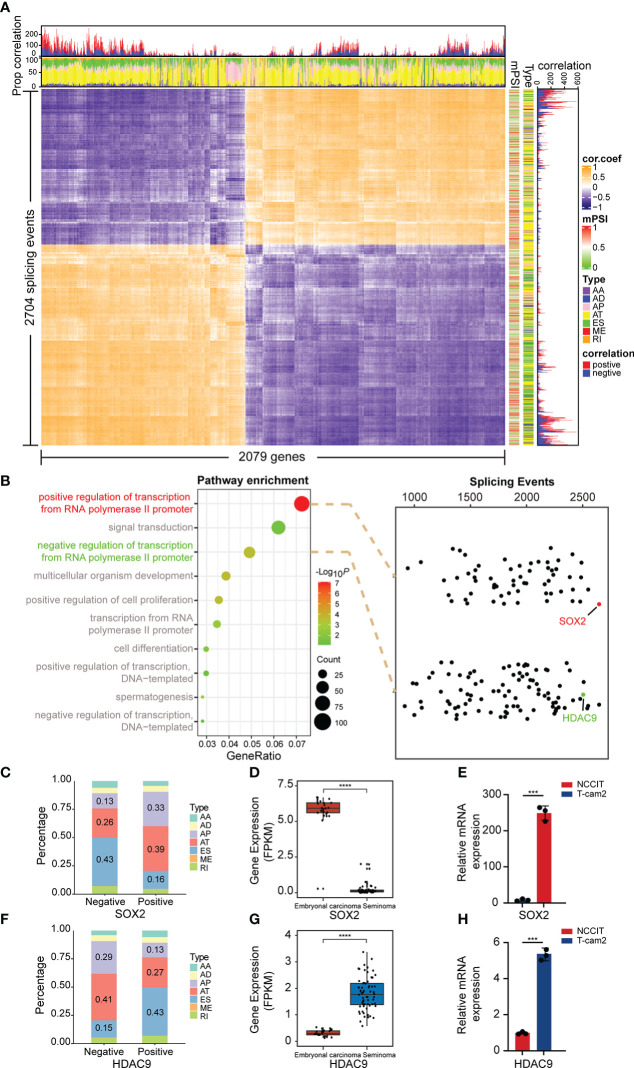
Correlation analysis of splicing and expression. **(A)** Heatmap demonstrated the correlation between differentially expressed genes and differentially spliced events (with FDR<0.05, delta PSI>0.1). **(B)** Spearman correlation analysis was performed of all genes in the “positive/negative regulation of transcription from RNA polymerase II promoter” pathway with DASE s. **(C, F)** Percentage of positively and negatively correlated splicing types in AS events associated with SOX2 and HDAC9. Negative: Negative correlated AS events; Positive: Positive correlated AS events. **(D, G)** Gene expression of SOX2 and HDAC9 in embryonal carcinoma and seminoma from TCGA. **(E, H)** Gene expression of SOX2 and HDAC9 were validated in TGCT cell lines. ****P < 1e-04, ***P < 0.001.

To broadly explore the potential relationship between splicing and expression in the above two subtypes, Spearman correlation analysis was further performed on DAS and DEG. The results suggested that the proportion of positive and negative correlations was almost equal, and the related splicing types were mainly AT and ES, indicating that AS and expression may interact mainly through these two ways (**Figure 4A**). Therefore, we considered whether some genes also have regulatory roles in AS. According to the results analyzed in **Figure 1C**, we observed that most genes were enriched in “positive/negative regulation of transcription from RNA polymerase II promoter” and other transcriptional regulation related pathways. Subsequently, we conducted correlation analysis between all genes in these two pathways and DASEs. AS events with weak correlation were excluded, and all splicing events with value of correlation coefficient |r| ≥ 0.4 were selected. SRY-Box Transcription Factor 2 (SOX2), a member of the SOX transcription factor family, regulates embryonal development through transcription and was observed to be the most DASEs-related gene in the “positive” pathway. In addition, we found that Histone Deacetylase 9 (HDAC9), which plays a key role in transcriptional regulation and cell cycle progression, was significantly associated with DASEs in the “negative” pathway (**Figure 4B**). Moreover, by analyzing all AS events, we revealed that of all 2674 SOX2 related AS events, 1646 were positively correlated and 1028 were negatively correlated. The predominant splicing types in negative correlations were ES and AT, while AT and AP were the main splicing types in positive correlation (**Figure 4C**). Conversely, of all 2502 AS associated with HDAC9, 957 were positively correlated and 1545 were negatively correlated. The main splicing types in the negative correlation are AT and AP, while in the positive correlation it is ES and AT (**Figure 4F**). Moreover, data from TCGA showed that SOX2 was significantly overexpressed in embryonal carcinoma cells (**Figure 4D**), while HDAC9 expression was increased in seminoma cells (**Figure 4G**). Interestingly, this result was confirmed in TGCT cell lines *via* qPCR (**Figures 4E, H**).

### Validation in TGCT cell lines

**Figure 5:**
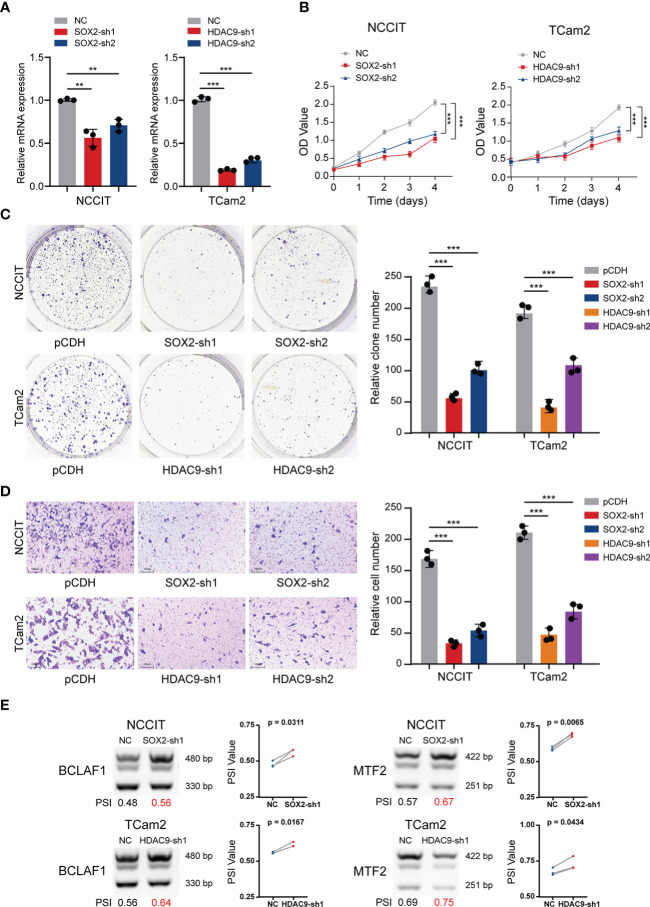
Validation experiments of SOX2 and HDAC9 in TGCT cell lines. **(A)** The efficiency of TGCT cell lines stably silencing SOX2 and HDAC9 was validated by RT-PCR. **(B, C)** CCK-8 and Colony formation assays were performed in TGCT cell lines. **(D)** Transwell migration assay was applied in TGCT cell lines. **(E, F)** Semi-quantitative PCR was performed to detect the alternation of PSI value of DAS events in cell lines after silencing SOX2 and HDAC9. ***P < 0.001, **P < 0.001.

Given the strong correlation of SOX2 and HDAC9 with AS and their key regulatory roles in transcription, further experimental verification was performed. TGCT cell lines (including NCCIT and TCam2) stably silencing SOX2 or HDAC9 were constructed respectively and their efficiency were validated (**Figure 5A**). CCK-8 and clone formation assays revealed that cell proliferation was significantly decreased after SOX2 and HDAC9 knockdown in NCCIT and TCam2 cells, respectively (**Figures 5B, C** **)**. In addition, Transwell migration indicated that silencing these two genes significantly inhibited cell migration and invasion (**Figure 5D**). Combined with the previous description, we hypothesized whether the alternation of these two key genes in TGCT cell lines were also associated with the regulation of AS. Therefore, semi-quantitative PCR was performed to detect AS of related genes in the cell lines after interference. As shown in **Figure 2G**, the proportion of BCLAF1 long splicing-​​isoform in TCam2 cells was significantly higher than that in NCCIT cells, but after SOX2 and HDAC9 were knocked down, the long isoform of BCLAF1 were significantly increased in both cell lines (**Figure 5E**). Meanwhile, the long splicing-​​isoform of another gene, MTF2, was increased in SOX2 knockdown NCCIT cells and HDAC9-interfered TCam2 cells (**Figure 5E**). As we described in **Figure 2G**, AS in exon 11 of BCLAF1 and exon 8 of MTF2 lead to differences in the proportion of long and short isoforms in NCCIT and TCam2 cell lines. After SOX2 and HDAC9 interference, exon AS of BCLAF1 and MTF2 was affected, resulting in the ratio of long and short isoforms to change again in TGCT cell lines. The effect for this result may be that we analyzed in **Figure 3B**. AS may increase the ratio of NMD transcripts, change the proportion of long and short transcripts, or even affect the sequence and structure of proteins. Interestingly, BCLAF1 and MTF2 are transcription factors (TFs), suggesting that TFs itself is also regulated by AS. Such changes may further regulate the function of genes and even influence the occurrence and progression of multiple diseases (46, 47).

### Differences of somatic mutation between embryonal carcinoma, mixed germ cell tumor and seminoma

**Figure 6:**
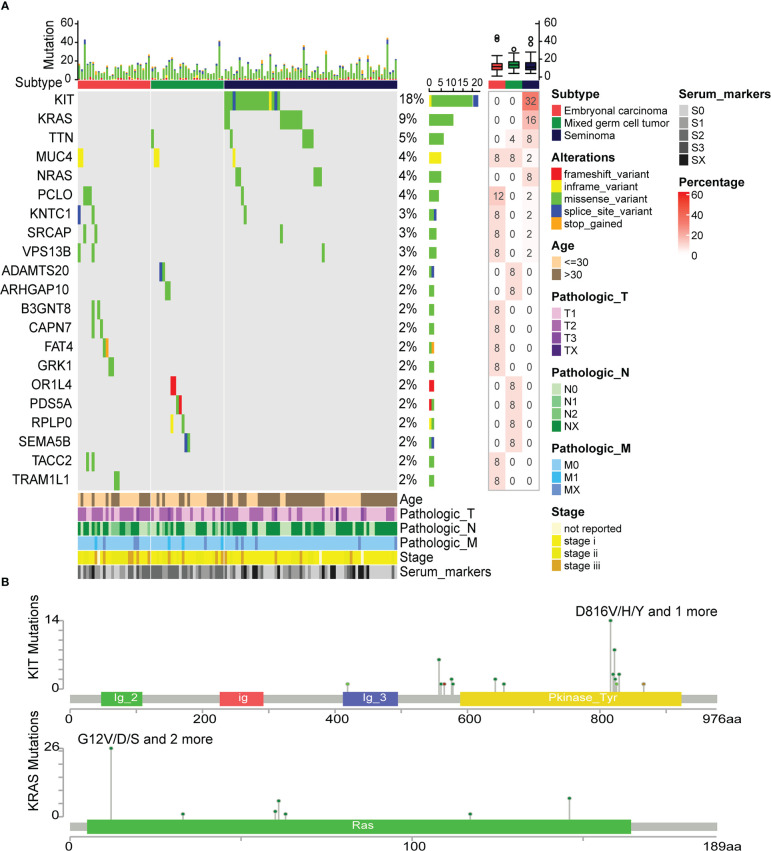
Differences in somatic mutations between TGCT subtypes. **(A)** The somatic mutations between embryonal carcinoma, mixed germ cell tumor and seminoma, and the percentage of mutated genes in each group (frequency>1%). **(B)** The mutation site map of high-frequency mutation genes (KIT and KRAS).

To understand the effect of somatic mutations on TGCT subtypes, mutation analysis was performed. Twenty-one genes with mutation frequency greater than 1% among 114 TGCT samples were included in this analysis (**Figure 6A**). At the same time, we found that there are six genes with mutation frequency greater than 3% (high-frequency mutation), including KIT (18%), KARS (9%), TTN (5%), MUC4 (4%), NRAS (4%) and PCLO (4%). KIT, KARS and NRAS observed at very high mutation frequency were mainly occurred in seminoma. Mutations in ADAMTS20, ARHGAP10, OR1L4, PDS5A, and RPLP0 were only found in mixed germ cell tumors, while mutations in B3GNT8, CAPN7, FAT4, GRK1, TACC2 and TRAM1L1 were only observed in embryonal carcinoma, and their mutation frequency was around 2%. TTN mutation existed in mixed germ cell tumor and seminoma, and all of them were missense variant. The mutation of MUC4 was found in the three subtypes, and all appeared as inframe variant. The bar plot above showed the number of mutation types in each patient. Overall, missense variant account for the vast majority of all mutation types, and stop gained was the least, which only occurred in the FAT4. Furthermore, the mutation percentage of each gene in the group was shown in the heatmap on the right. Since mutation percentages of KIT and KARS within the group were the highest, the cBioPortal website was used to analyze the mutation sites of them (**Figure 6B**). The mutation sites of KIT were primarily located in the tyrosine kinase domain. These mutations affect the signal transduction of the cell by changing the activity of the kinase, affecting normal germ cell development and increasing the incidence of seminoma (48). KRAS mutations mainly occur in the RAS domain, and many mutations lead RAS to continuously stimulate the downstream pathways, leading to the proliferation of cancer cells (49). Recently, Hofmannet.al discovered that the small molecule BI-3406 binding to the catalytic domain of SOS1 can reduce the formation of GTP-loaded RAS, thereby limit the proliferation of cancer cells driven by KRAS (50). This discovery provides a new theoretical basis for the study of targeted therapies for cancers with KRAS mutations.

## Discussion

In this study, we systematically analyzed the expression, AS and somatic mutation data of patients with three main subtypes of TGCT from TCGA, and the differences between embryonal carcinoma and seminoma were emphasized. The differential expression and DAS events genes between the two groups were determined, and GO pathway enrichment was performed to understand their functions. Furthermore, the key genes with research value or clinical concern have been independently analyzed and validated in TGCT cell lines. Finally, we further identified splicing-regulated genes and demonstrated their valuable role on TGCT proliferation and invasion through a series of functional experiments.

Through the unremitting efforts of clinicians and researchers, the therapies of TGCT have achieved very significant results. However, an increasing number of patients are facing reduced quality of life due to tumor recurrence and complications of radiotherapy and chemotherapy. Many researches on TGCT gene expression have been reported recently. Chang et al. evaluated the expression patterns of cancer-testis (CT) genes of TGCT patients in TCGA, and confirmed the role of CT genes in the prognosis of TGCT (51). By retrospectively analyzing the characteristics of patients with stage I TGCT, Lewin identified the differential gene expression profile between patients with relapsed and non-relapsed TGCT (52). Mallik and his colleagues combined analysis of gene expression and methylation in seminoma and non-seminoma, providing a co-regulation research perspective (13). However, the expression differences of TGCT subtypes have not been fully explored. As an important post-transcriptional regulatory mechanism, alternative splicing (AS) occurs in the normal physiological process of most human genes, but abnormal splicing is a potential cause of many diseases, including cancers (53, 54). The role of abnormal AS in tumor progression, recurrence, and drug resistance has been observed in previous studies, and the role of AS in the progression of TGCT has also been reported (55). However, the splicing differences between the different subtypes of TGCT need to be further studied. Furthermore, in addition to abnormal AS, the accumulation of somatic mutation in characteristic genes has been observed to be a significant cause of cancer. Somatic mutations can be divided into mutations that result in selective growth advantages (drivers) and mutations that do not result in selective growth advantages (passengers) (56). Clinically, most drivers can generate carcinogenicity by regulating key small molecule enzymes or binding to cell signaling receptors (57). Studies on somatic mutations of TGCT have also been reported (58, 59), but comparative analysis of TGCT subtypes has been rarely understood.

We comprehensively analyzed the differential genes for expression, AS and mutation among TGCT subtypes, and performed GO pathway enrichment analysis and validation experiments. Among expression-related genes, CDX4 and FOXD3 were significantly overexpressed in embryonal carcinoma, whereas CUX2 and RPS6KA5 were increased in seminoma (**Figures 1D, E** **)**. They are both important molecules in transcriptional regulation and have been shown to be involved in the progression of various tumors (60–63). In the future, they are expected to become characteristic molecules of embryonal carcinoma and seminoma to serve the clinic. Since we only displayed genes with significant differences, many other genes that have not been paid attention to deserve further exploration. The differences and regulatory mechanisms of AS are complex and various, and once AS happens, differences may occur (64). Therefore, the volcano map of DAS was almost symmetrically distributed (**Figure 2B**). The regulation of transcripts by splicing is also multifaceted, and several different splicing outcomes are shown in **Figure 3A**. The occurrence of AS in one exon may not affect the expression of the entire transcripts, but probably have a corresponding impact on the function it encodes. For example, Type II CAAX prenyl endopeptidase Rce1-like (RCE1), a member of the membrane-bound super protein family of Type II CAAX prenyl endopeptidase, is the functional domain of RCE1 for exon skipping. It is related to metal-dependent enzymes and may have a strong correlation with protein modification and secretion (65). The functional domain where exon splicing occurs on the first transcript of MRPL33 is Ribosomal protein L33. As a member of the Zinc-binding ribosomal protein superfamily, Ribosomal protein L33 can interact with other ribosomal subunits and is indispensable for ribosomal biogenesis and subunit connection (66). And MRPL33 has two different transcription variants, MRPL33-L and MRPL33-S, which have opposite effects on the growth and apoptosis of cancer cells (67, 68). On the one hand, AS lead to changes in the length of the transcript, thereby affecting its stability and function of the encoding gene. On the other hand, we believed that AS of a certain gene may lead to the degradation of transcripts and affect the mRNA expression of the gene, resulting in significant differences between TGCT subtypes. It is worth noting that we mainly focus on the CDS (Coding sequence) region of the transcript, while the 3’ and 5’ UTR (Untranslated Regions) regions still have a large number of AS events. Although they are removed during normal post-transcriptional modification, the occurrence of AS in these regions may affect the regulation of translation. For example, the location of AS in the 5’ UTR region may be involved in the translation regulation of spliced-​genes. Splicing events located in the 3’ UTR region can affect mRNA stability and degradation rate by changing the length or sequence of the region, thus altering gene expression levels. Somatic gene mutation is also a crucial driver of tumorigenesis and progression. Many studies have reported the observation of KIT, KRAS and NRAS gene mutations in TGCT (69, 70). Based on this, the mutation differences between subtypes were further analyzed. As described in our results, mutation that only occurs in embryonal carcinoma (B3GNT8, CAPN7, FAT4, GRK1, TACC2 and TRAM1L1) or seminoma (KIT, KARS and NRAS) may be valuable molecular markers and therapeutic targets that need to be considered clinically. Mutations in MUC4 were found in all three groups, suggesting that basic medication targeting MUC4 may be beneficial for patients when their clinical subtypes are unknown. In addition, missense mutations account for the most of all mutation types, and almost all genes have been observed to contain missense mutations. The mechanism of TGCT missense mutation and its related targets may be the potential focus of future clinical research.

The limitations of this study are also worth noting. First, only three major subtypes of TGCT were analyzed, and the remaining cases of yolk sac tumor and teratoma were too small to be compared. Therefore, the sample size needs to be further expanded. Second, we only analyzed the patients of TGCT in the TCGA database, which may have certain limitations. It may be more convincing to further combine the multi-center database in the future. Third, for the analysis of splicing and expression correlation, we mainly illustrate by exon skipping, other types of splicing may be more complicated, and further in-depth analysis is required to clearly understand the reasons and mechanisms of the differences.

## Conclusions

The result of analysis increased our understanding of the differences (including gene expression, alternative splicing, and mutations) between TGCT subtypes. For the first time, our research indicates a clear correlation between AS events and gene expression in TGCT, and the possible reasons were analyzed. In conclusion, our study provides a molecular basis for the clinical diagnosis and precision therapy of TGCT, which can serve as a potential marker for future clinical diagnosis and therapeutic targets.

## Data availability statement

The datasets generated for this study can be found in the https://xenabrowser.net, and http://bioinformatics.mdanderson.org/TCGASpliceSeq/index.jsp.

## Ethics statement

Ethical review and approval were not required for the study on human participants in accordance with the local legislation and institutional requirements. Written informed consent for participation was not required for this study in accordance with the national legislation and the institutional requirements.

## Author contributions

XY and HZ designed the study. XY, HZ and CD collected and analyzed the data. XW, BL and HL prepared the figures and tables. XY, HZ and YZ wrote the manuscript. XYY and YJZ revised the manuscript. All authors contributed to the article and approved the submitted version.

## Supplementary material

Supplementary Figure 1 The differentially expressed genes and spliced genes in GO enrichment pathways. **(A)** The number of up regulation and down regulation of differentially expression genes in each GO enrichment pathways. **(B)** The number of up regulation and down regulation of differentially spliced genes in each GO enrichment pathways.

Supplementary Figure 2 Expression of differential genes in embryonal carcinoma and seminoma. **(A)** Enrichment heatmap of differentially expression genes in the “positive regulation of cell proliferation” pathway. **(B-E)** Expression of several clinically relevant genes in two subtypes. ****P*<0.001.

Supplementary Figure 3 Patterns of splicing types and their numbers in all AS events. **(A)** The descriptive pattern of seven splicing types. **(B)** The bar graph showed the number of all AS events in seven splicing types from TGCT cohort.
