# Supplementary material for: Comprehensive characteristics of pathological subtypes in testicular germ cell tumor: Gene expression, mutation and alternative splicing
Source: Front Immunol. 2023 Jan 13;13:1096494. doi: 10.3389/fimmu.2022.1096494 (PMC9883017; doi:10.3389/fimmu.2022.1096494)
Supplement: Supplementary file 1 [file Table_1.docx]

**Table S1. The primers used in qRT-PCR and sqRT-PCR.**

| **Primer name** | **Primer sequence (5′ - 3′)** |
| --- | --- |
| GAPDH | F: ACAACTTTGGTATCGTGGAAGG  R: GCCATCACGCCACAGTTTC |
| CUX2 | F: CGAGACCTCCACACTTCGTG  R: TGTTTTTCCGCCTCATTTCTCTG |
| CDX4 | F: CCGATGCCAGCCTCCAATTT  R: CTGTGCCCATTGTACTAGACG |
| FOXD3 | F: ACTCTGCCTCTCCCCAATTT  R: CCATCCCCACGGTACTAAGA |
| HDAC9 | F: GAATCCTCAGTCAGTAGCAGTTC  R: GGGGCAAAACCGAAGTCTCAT |
| SOX2 | F: TACAGCATGTCCTACTCGCAG  R: GAGGAAGAGGTAACCACAGGG |
| RPS6KA5 | F: AGGCAGTCGCCATTTTTGGTA  R: TCTCCAACATAAATCTGCACCTC |
| ARHGAP17(ES) | F: AGGTGGAATTTAATGTATCAGAAGCA  R: CCTGCAGCATTGTGAGGTTG |
| BCLAF1(ES) | F: ACCTTCTTCTCCCAGTTCTCG  R: TCCTTGCGTCTGTCCTTCTTT |
| CDC25B(ES) | F: GTTTGAACAGGCCATCCAGG  R: GGGCTTCCATGGCATCTTGA |
| CDK5(ES) | F: TCCTGCACAGCGACAAGAAG  R: TACAGCTTGGCCCCAAAGAG |
| ETNK2(ES) | F: CTGGTGGACCGGGAGAATG  R: CTTTGATGCTGTCATAGATGATATTCT |
| MTF2(ES) | F: GCACACCTATGCCTTTACAACC  R: GTATATGGGCCAGGTGGACG |
| SOX2(sh1) | F: GATCCGCTCATGAAGAAGGATAAGTCTCGAGACTTATCCTTCTTCATGAGCGTTTTTG  R: GGCCCAAAAACGCTCATGAAGAAGGATAAGTCTCGAGACTTATCCTTCTTCATGAGCG |
| SOX2(sh2) | F: GATCCAGCTCGCAGACCTACATGAACTCGAGTTCATGTAGGTCTGCGAGCTGTTTTTG  R: GGCCCAAAAACAGCTCGCAGACCTACATGAACTCGAGTTCATGTAGGTCTGCGAGCTG |
| HDAC9(sh1) | F: GATCCAAACTGCTTTCGAAATCTATCTCGAGATAGATTTCGAAAGCAGTTTGTTTTTG  R: GGCCCAAAAACAAACTGCTTTCGAAATCTATCTCGAGATAGATTTCGAAAGCAGTTTG |
| HDAC9(sh2) | F: GATCGAGCAGTTAATAGGCTTTAAACTCGAGTTTAAAGCCTATTAACTGCTCTTTTTG  R: GGCCCAAAAAGAGCAGTTAATAGGCTTTAAACTCGAGTTTAAAGCCTATTAACTGCTC |
