# Supplementary figures and images for: Comprehensive characteristics of pathological subtypes in testicular germ cell tumor: Gene expression, mutation and alternative splicing
Source: Front Immunol. 2023 Jan 13;13:1096494. doi: 10.3389/fimmu.2022.1096494 (PMC9883017; doi:10.3389/fimmu.2022.1096494)

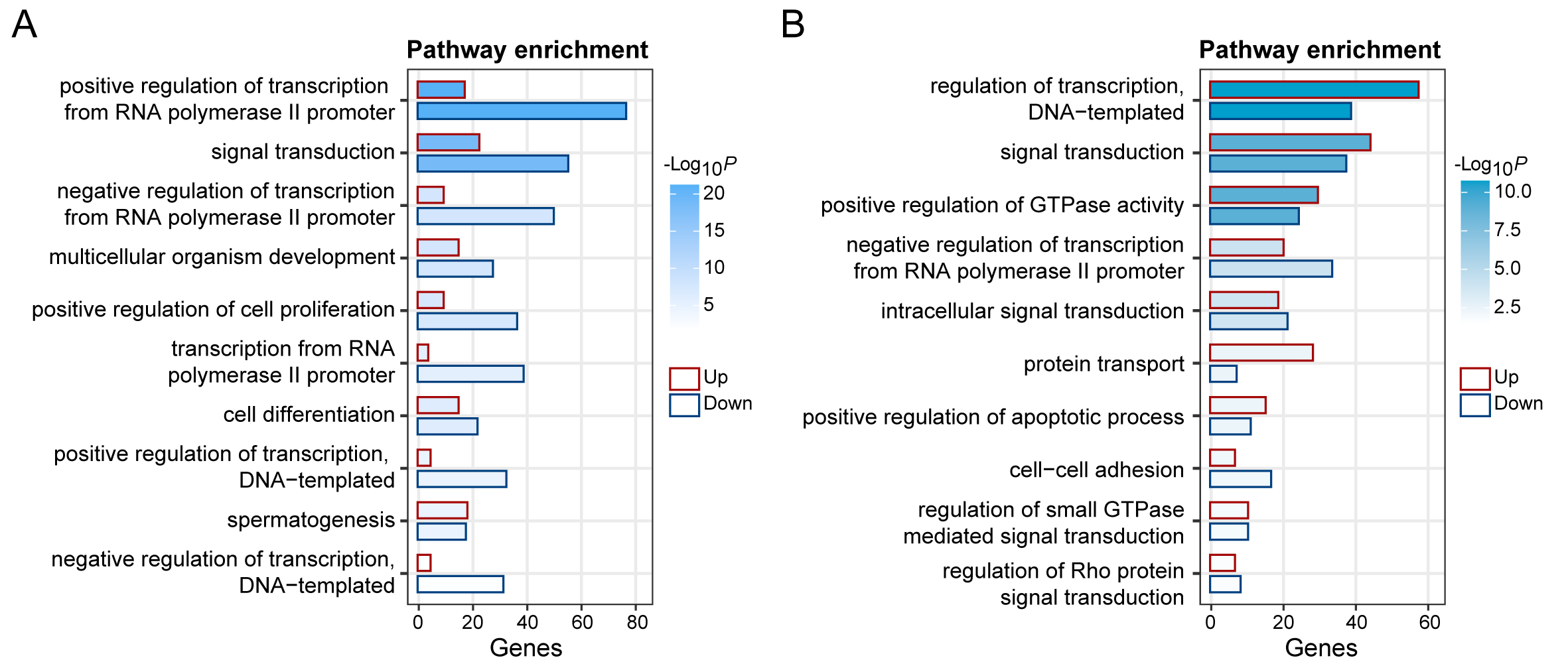

Supplement: Supplementary Figure 1 — The differentially expressed genes and spliced genes in GO enrichment pathways. (A) The number of up regulation and down regulation of differentially expression genes in each GO enrichment pathways. (B) The number of up regulation and down regulation of differentially spliced genes in each GO enrichment pathways. [file Image_1.tif]

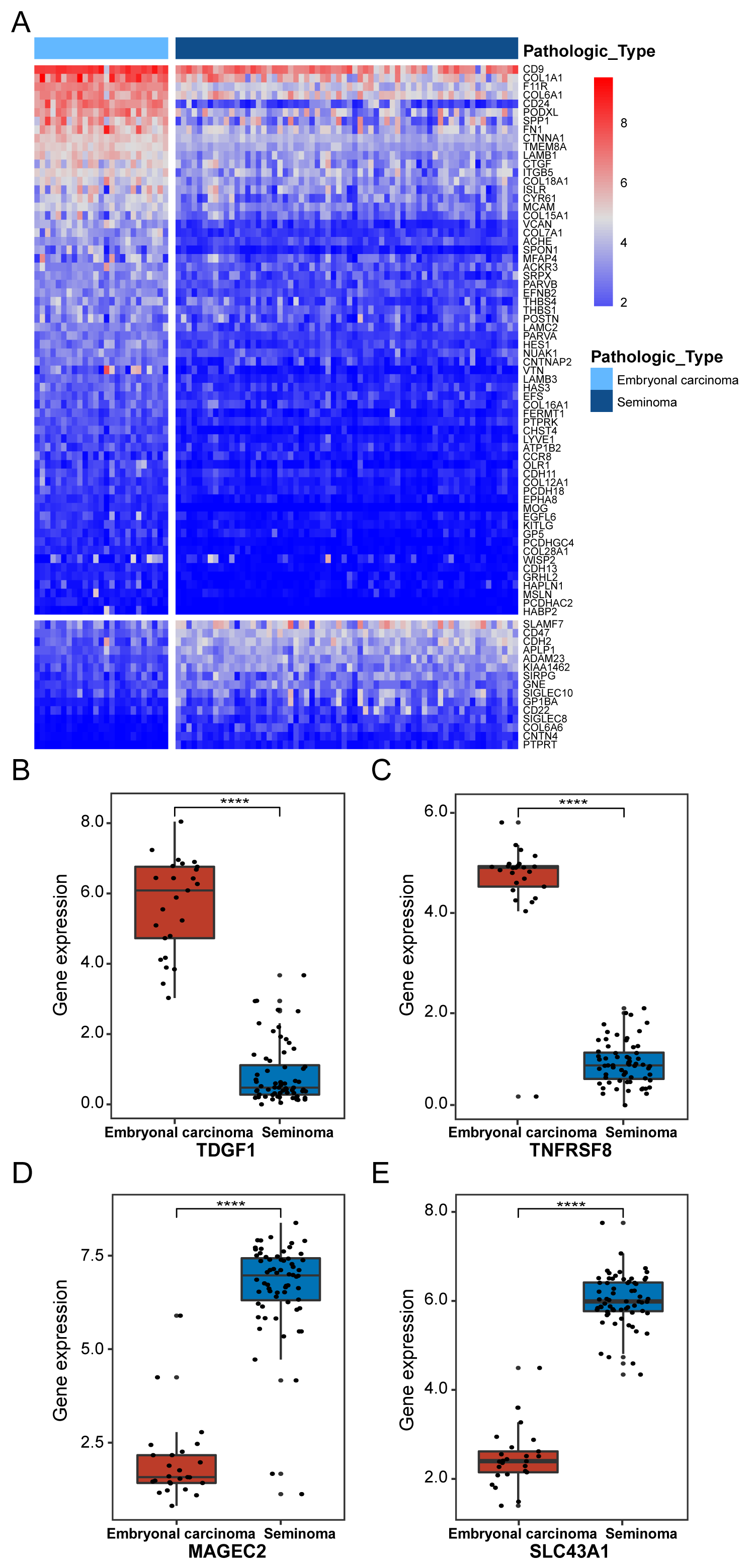

Supplement: Supplementary Figure 2 — Expression of differential genes in embryonal carcinoma and seminoma. (A) Enrichment heatmap of differentially expression genes in the “positive regulation of cell proliferation” pathway. (B-E) Expression of several clinically relevant genes in two subtypes. ***P<0.001. [file Image_2.tif]

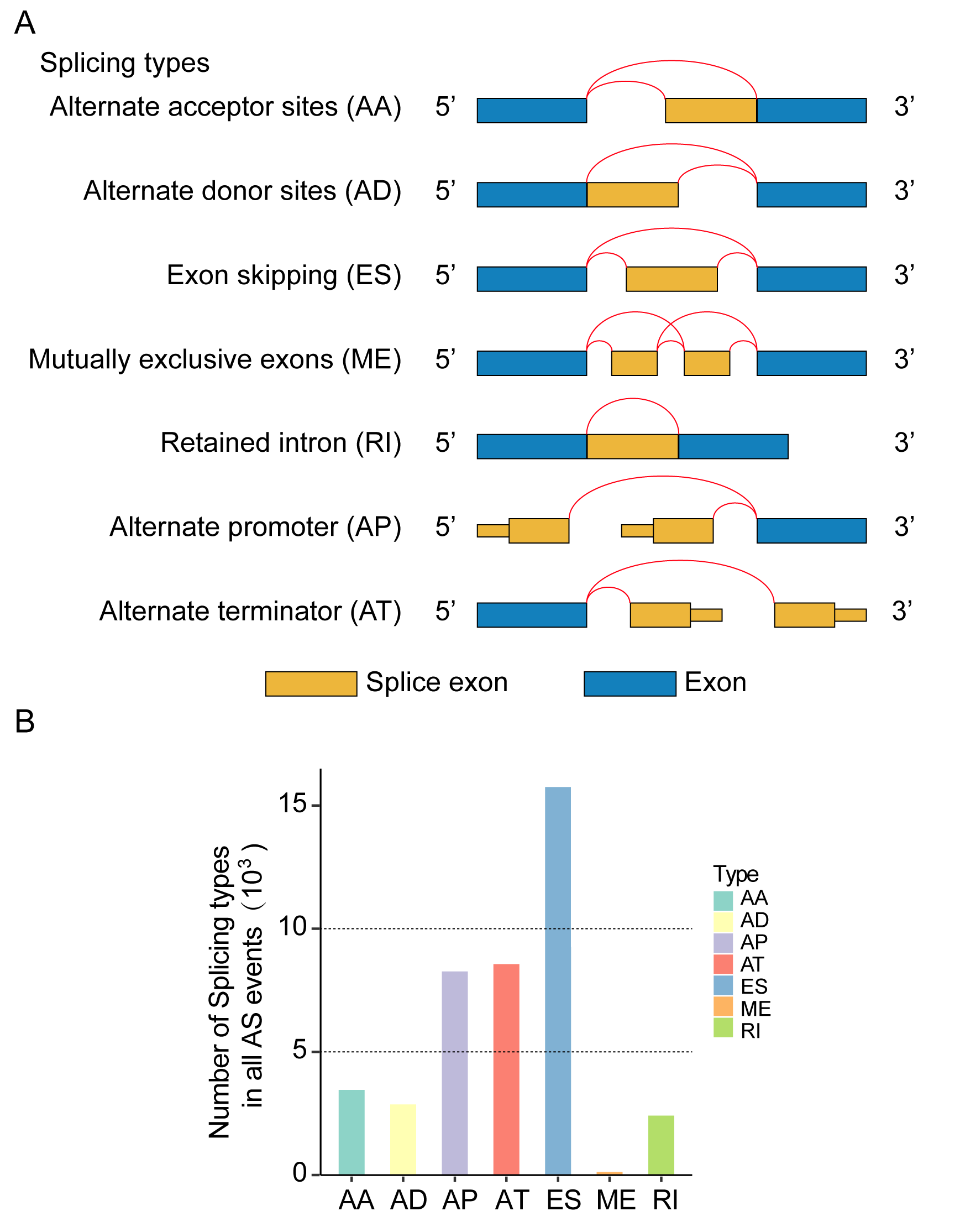

Supplement: Supplementary Figure 3 — Patterns of splicing types and their numbers in all AS events. (A) The descriptive pattern of seven splicing types. (B) The bar graph showed the number of all AS events in seven splicing types from TGCT cohort. [file Image_3.tif]
